# Empirical Study of Large-Scale HLA Simulation of Parallel Region-Matching Knowledge Recognition Algorithm Based on Region Matching

**DOI:** 10.1155/2022/1514396

**Published:** 2022-04-11

**Authors:** Guohua Zhu, Haizhou Wang

**Affiliations:** School of Artificial Intelligence, Jianghan University, Wuhan 430056, Hubei, China

## Abstract

Most of the existing region-matching algorithms need to match all regions, resulting in a waste of computing resources, increasing the cost of simulation technology and data redundancy, and resulting in the reduction of network data stream transmission efficiency. This paper presents a parallel region-matching knowledge recognition algorithm. Combined with the shortcomings of existing matching algorithms, a simulation technology is constructed to realize the parallel matching of multiple regions in HLA distributed simulation. The algorithm can realize the parallel matching calculation of multiple changed regions in one simulation. At the same time, the basic idea based on mobile intersection is adopted in the matching calculation, and the historical information before and after the region range is moved is used. The matching is limited to the moving interval, and the moving crossover theory is applied to the matching calculation to realize the relevant historical information before and after the region. Simulation results show that the parallel region-matching knowledge recognition algorithm can support HLA distributed simulation evaluation. In the matching calculation, the basic idea based on moving intersection is adopted, and the matching is limited to the moving interval by using the historical information before and after the region is moved, which reduces a large number of irrelevant calculations. Theoretical analysis and experimental results show that the algorithm is particularly suitable for the application needs of building large-scale distributed simulation based on multi-core computing platform.

## 1. Introduction

With the continuous development of social economy, engineering construction projects have started to be implemented one after another [1, 2]. For large-scale construction, relying only on traditional design drawings may not fully meet the needs [3–5]. Therefore, experts within the industry have introduced computer simulation technology to further shorten the corresponding period, improve the corresponding training quality, and save the corresponding expenditure. It has been widely applied in many fields [6–8]. There are also many in-depth explorations in China and other countries, such as proposing corresponding high-level architecture (HLA), creating corresponding distributed simulation based on a flexible and customized architecture, and realizing the interoperability and reusability of various modules and simulation bodies. For HLA, it mainly includes rules, interface specifications, and object templates. In the specific simulation process, distributed simulation can be regarded as a complete dataset, and each simulation component can be called a member [9, 10]. In the existing HLA simulation process, if the relationship between the simulation models is coupled, it will cause the corresponding system to lack a certain degree of flexibility and cause the overall simulation efficiency to decrease [11, 12]. Therefore, if the dynamic and orderly allocation of resources is to be achieved, the simulated client terminal and the server need to be effectively separated. Some scholars integrate grid technology and simulation technology to improve the above shortcomings and use the separation of the simulated client terminal and the server to achieve dynamic adjustment [12, 13]. Such a fusion method has certain advantages, such as dynamic adjustment and automatic allocation of data resources, full-process support for the entire life cycle of simulation, strong support for security performance, adaptability to dynamic changes of the grid, automation of resource selection, performability of operating data, automatic collection of simulation results, enhancement of fault tolerance performance, and so on [14, 15].

As the scale of simulation continues to increase, the efficiency of simulation calculation has declined. For simulation entities with a huge amount of data, they all have considerable communication or connection with each other, and these data volumes often show exponential changes or growth. Especially in a specific practical application environment, how to achieve effective data distribution and data simulation in such a situation is a problem worthy of in-depth study [16, 17]. The first is to filter a large amount of data based on related data filtering methods, aiming to reduce the transmission and reception of redundant data during the data simulation operation, thereby reducing the feasibility and data flow of the unit to receive the corresponding redundant data [18–20]. Secondly, as the number of simulation entities increases, HLA distributed simulation experiments will have a large amount of data movement during each simulation experiment [21, 22]. In order to ensure the effectiveness and rationality of data distribution, it is necessary to perform reasonable overlap matching on the overlapping relational areas. Meanwhile, the more the number of entities in the simulation entity, the greater the difficulty of matching. Therefore, the matching algorithm directly determines the efficiency of the simulation and also affects the scalability of corresponding simulation. In view of these needs and deficiencies, based on the parallel region-matching knowledge recognition algorithm, the corresponding simulations are used to achieve parallel matching of multiple regions through combining the business logics of HLA distributed simulation, and the theory of mobile intersection is used in matching calculations to achieve the historical information related to forward or backtracking region, which is limited to a fixed threshold, reducing invalid operations and aiming to improve the effectiveness of HLA distributed simulation.

### 1.1. HLA Distributed Evaluation Method

#### 1.1.1. Concepts Related to Regional Matching

**Figure 1:**
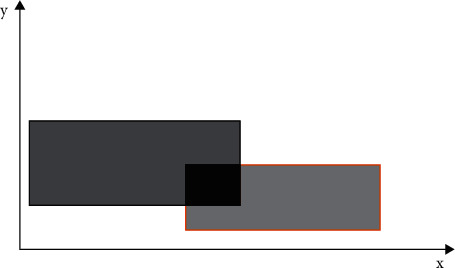
Description of overlapping regions in a two-dimensional interest space.

In view of area matching, in the specific simulation specification, the management of data distribution is based on the corresponding area. The data collector or producer uses the effective designation of the fixed area to achieve data production, and the data user uses search for a specific area to achieve reception of the data. Take a specific area overlap as an example, as shown in Figure 1.

**Definition 1 .**

Dimension: the so-called dimension is based on a specific named interval, which is essentially a non-negative interval. The downward limit is 0, and the upward limit varies according to the specific dimensions. Its value needs to be defined in the corresponding dimension table.

**Definition 2 .**

Range: it is different from dimension. It is a continuous integer half-open interval and is a sub-dataset of related dimensions. It is specifically defined by an ordered integer. The specific first number can be considered as the downward limit of the range, and the second integer is the upward limit.

**Definition 3 .**

Region description: it is a specific range dataset, that is, the corresponding dimension described by the range contained in the area is used. For each dimension described in a specific area, it can only have one range.

**Definition 4 .**

Region realization: the so-called region realization is to realize attribute update, interactivity, data query, etc. through specific associations.

**Definition 5 .**

Region: it is the general term for the description and realization of the region.

**Definition 6 .**

Update region (publish region): The parallel area mainly realizes the real-time update of data to meet the conditions of data query.

#### 1.1.2. Parallel Region-Matching Knowledge Recognition Algorithm

The essence of the so-called region-matching algorithm is to determine whether there is a corresponding overlap in the individual interest units of the region, and the specific matching efficiency and accuracy determine the relevant efficiency of specific interest matching [23, 24]. The specific typical matching algorithm can be divided into direct matching, grid matching, mixed matching, classification matching, mobile intersection matching, etc. according to the matching principle or method [25, 26].

For direct matching, its essence is to use a separate data structure to update the range of data and the specific range of query subscriptions. When users query specific data, the algorithm in this paper will identify the corresponding data information according to specific conditions and then calculate the overlapping area with the identification. This method is relatively simple, not requiring additional information, and can ensure accurate matching. However, this algorithm has a relatively large computational complexity. For a large number of areas of large-scale simulation, this algorithm is difficult to adapt to this simulation calculation [27, 28].

For grid matching, the essence is to use the simulation system to divide the full-dimensional regular grid in advance and set the corresponding data-related channel for each network logic, especially for the specific calculation of the regional boundary to determine the correlated network covered by the region, so as to clarify the relevant channels of data transmission. If the data update area and the query data area have the same unit, then grid matching can be performed. The complexity of the network matching algorithm is relatively simple compared with direct matching, but network matching often generates redundant links and requires manual data filtering. From the overall effect, the specific network matching algorithm is difficult to reconcile in the specific matching speed and accuracy to a certain extent, and the size of the grid affects the efficiency of the overall algorithm.

In regard to the limitations of the direct matching and network matching algorithms, some scholars have proposed a related algorithm of hybrid matching, which uses specific network matching to achieve one-to-one mapping of all regions to a specific network and realizes the exact matching simple compared with specific network. On the one hand, this hybrid algorithm can ensure the accuracy of matching but also reduce the complexity of the entire direct matching algorithm. However, consistent with the network algorithm, the integrity of the hybrid matching algorithm still needs to be restricted by the size of the network.

For the classification matching algorithm, its essence is to use all areas of the system to perform multi-dimensional projection sorting, realize the overlap analysis of two or more dimensions on the projection, and indicate that these ranges have a certain intersection and overlap. The complexity of the classification matching algorithm is relatively moderate, that is, the classification matching algorithm can be used to complete the overlap judgment of the corresponding area at one time. This judgment can realize the misalignment of the operation and the improvement of the matching efficiency. However, because parallel matching recognition is to effectively detect the corresponding region, in the actual simulation process, if some recognition regions change, the system will be recalculated, resulting in the decline of recognition rate.

The specific mobile matching is used to achieve dynamic matching, the essence of which is that only part of the region of the large-scale simulation system changes, so it only needs to change according to the information before and after the area change to realize the information movement of the matching calculation and the effective change of matching range, while effectively reducing the amount of specific matching calculations. During the process of mobile matching, the specific algorithm matching degree only needs to be related to the number of change regions and has nothing to do with the total number of regions of the entire system. Therefore, it can be seen that mobile matching is not only dynamic but also accurate and efficient.

Therefore, it is necessary to use multi-core CPUs for actual processing. When the computing platform becomes multi-core and uses distributed interactive simulation on a larger scale, specific high-performance parallel computing can be used to improve specific simulation performance and achieve support for complex systems. Although the above matching algorithm causes certain restrictions and limitations to large-scale simulation calculations, there are also certain limitations, which are restricted by the bottleneck of computational complexity. Therefore, it is worthy of in-depth exploration and research to realize efficient parallel computing on a multi-core, distributed computing platform.

**Figure 2:**
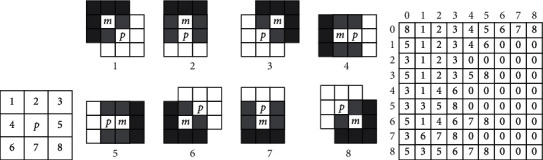
Pixel 8 neighborhood priority order, scan template, and two-dimensional array. (a) Scan-line order. (b) Scanning templates for p's different neighborhoods. (c) Array of the scanning templates.

First, a specific domain scanning method is used to collect data, and the specific sequence is shown in Figure 2. When there is overlap between *p* and the surrounding area pixels, it is necessary to fully consider the scanning speed to avoid repetitive scanning.  Figure 2(b) shows a specific scanning scheme, in which the shaded parts of the diagonal lines are overlapping areas. During scanning, these parts do not need to be scanned repeatedly, and only important grid parts are scanned for specific pixel point judgment. [1, 8] is used to determine the scan template.

A specific two-dimensional array is used as a scanning template, each specific row is used as a scanning template, each column of data is used as the number of scanning points included, and the rest are filled with 0. When a specific pixel point is scanned, the adjacent points need to be assigned to the specific periphery, and the circular labeling is realized according to the specific point.

The breadth-first method is used to search the parallel region-matching knowledge recognition algorithm. Assuming that the image is a two-dimensional image with *M* rows and N columns, the valueless represents black pixels, and value 1 represents white pixels. The specific steps are shown below.

1. First, create a specific tag array to indicate whether the pixels of the image have been processed. When the image is scanned, the array with tag attributes gives different tag values for each specific area.
2. The principle of left-to-right and top-to-bottom is used to effectively scan the image. When realizing a specific marked pixel scan, if the pixel is marked, it needs to be recorded for subsequent mark analysis.
3. When searching for the area at the starting point of the area, a fixed algorithm needs to be specifically called, and the label value of the area is set to a fixed value; when the function completes the specific call, the coordinates of all pixels in the area can be obtained.
4. Scan the image through *p*, repeat steps 2 and 3 iteratively, until the specific image is scanned, and the algorithm process ends.

**Figure 3:**
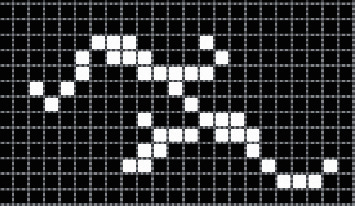
Binary graph of a single region.

On the basis of the above steps, the specific number of regions can be obtained, and the corresponding parameters can be used to mark each part of the region to achieve the specific mark value to obtain the corresponding region containing points. In the specific steps, it is necessary to implement the algorithm call of the connected region through the algorithm, and the single-region binary graph is shown in Figure 3.

**Figure 4:**
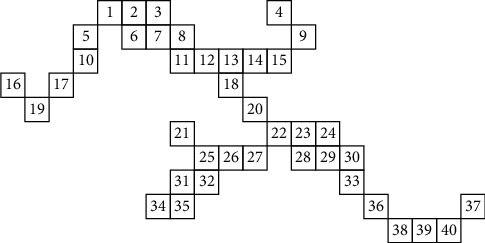
Pixel numbers given from top left to bottom right.

In order to realize the flow of the algorithm, the serial number is marked according to the specific search order. As shown in Figure 4, when the image is scanned to a specific value (1), the value of the corresponding array is recorded as 0, and it is taken as the starting point and ending point of the new region, the total number of corresponding regions is increased accordingly, and in the meantime, the value of the array is recorded as the corresponding array. On the one hand, it can indicate that this point has been scanned accordingly; on the other hand, it also belongs to certain region.

1. Use the corresponding queue to record the coordinates of the corresponding recording point and the scan mark.
2. The circular identification is performed for the corresponding queue; it is necessary to ensure that the queue is not null. When reading the point value at the head of the queue, it needs to be identified according to the specific scan template, and the inspection and marking are given in the corresponding order. Meanwhile, the marked points that are not scanned are appended to the queue through the corresponding array value, and then the scanned point is removed.

**Figure 5:**
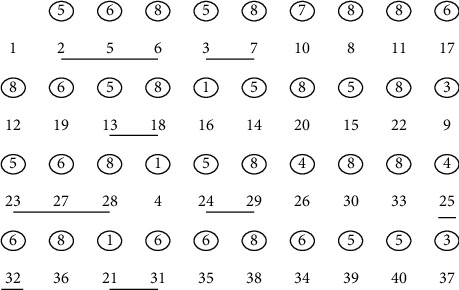
Points of the regional operation process.

The specific calling process is shown in Figure 5, the number in the circle represents the specific label of the scan template at a certain point, and the specific underlined point can be identified as the critical point of the same point. It can be seen from the running results of Figure 5 that if a different scanning sequence is used to scan the region of pixels, it is often unnecessary to traverse the surrounding points, and only 5 points need to be traversed at most. This scanning method effectively improves the operating efficiency. There is no specific mark conflict in the whole scanning process, and the final region will be scanned in the corresponding order from top left to bottom right.

As the computing performance of the computer has been significantly improved, in order to facilitate the use of the computer, make maximum use of computing power, use connectivity detection to analyze the effectiveness of each pixel, and analyze the specific connection relation between the pixels before and after. The specific region merging is realized through related parallel algorithms, and different algorithms are used to realize the different complexity of parallel algorithms in dealing with specific region overlaps. If a specific function is called directly, you can directly obtain the relevant computing performance of the computer and divide the image according to the computing power. If it is a two-core computing power, it is divided into two parts, and the region detection of the two parts is performed according to the above method. When the calculation is completed, the overlapping region is effectively scanned, and then the relevant regions are merged.

By setting the corresponding image size, using multiple computers for calculation, and using specific parameters to represent specific threads, formulas (1) and (2) are used to calculate the start lines and end lines of the thread.

(1) $$\begin{array}{c} starline=\left(threadNum-1\right)\times H/N, \end{array}$$

(2) $$\begin{array}{c} endline=threadNum\times H/N-1. \end{array}$$

HLA-distributed simulation data are collected by sensor equipment and stored in large capacity data storage equipment. After data processing, it is sent to the corresponding program for operation.

(3) $$\begin{array}{c} T\left(a,a_{1}\right)=\frac{\sum_{i=1}^{n}\left({\left(q_{i}-s\right)}^{2}\left(q_{i}-s-l\right)\right)\left(q_{i}-s^{2}\left(q_{i}-s-l\right)\right)}{\sqrt{\sum_{i=1}^{n}{\left(q_{i}-s^{2}{\left(q_{i}-s-l\right)}^{2}\right)}^{2}}+{\left(q_{i}-s^{2}{\left(q_{i}-s-l\right)}^{2}\right)}^{2}}, \end{array}$$

where *T*(*a*, *a*_1_) is the set of characteristic attributes of HLA distributed data and HLA distributed characteristic expression; *q*_*i*_ is the number of data features after classification for HLA distributed data; and *s* is the feature content of the HLA distribution. It is a numeric argument, a property specific to HLA distributed data. After identifying the characteristics of HLA distributed data, non-characteristic attributes must be removed. It can reduce the error and improve the speed when collecting. The redundant data removal formula is as follows:

(4) $$\begin{array}{c} L=\overset{⟶}{q}+\frac{\sum_{i=1}^{n}{\left(T\left(a,a_{1}\right)\right)}_{i}\left(q-e\right)}{\sum_{i=1}^{n}{\left[T\left(a,a_{1}\right)\right]}_{i}^{2}}, \end{array}$$

where *L* defines the removal benchmark and removes those that meet the benchmark; $\overset{⟶}{q}$ indicates the filter request to be used when removing; e represents an existing redundant data removal request. The features of the data can be obtained by filtering.

(5) $$\begin{array}{c} C=\beta \left(\overset{n}{\underset{i=1}{\cap }}e_{i}+\frac{\sum_{i=1}^{n}T\left(a,a_{1}\right)\cdot {\left(a,a_{1}\right)}_{i}d^{i\theta }}{\sum_{i=1}^{n}T{\left(a,a_{1}\right)}_{i}^{2}}\right)+\left(1-\beta \right)\left(q_{i}+\frac{\sum_{i=1}^{n}\left(T{\left(a,a_{1}\right)}_{i}-\bar{X}\right)\cdot T{\left(a,a_{1}\right)}_{i}^{2}}{\sum_{i=1}^{n}{\left(T\left(a,a_{1}\right)-\bar{X}\right)}^{2}}\right), \end{array}$$

where *α* represents the ownership value of the data feature and *β* represents the correlation coefficient of the balance factor. Assume input vectors *x*_*i*_ and label values *y*_*i*_. The softmax loss function expression used in this paper is

(6) $$\begin{array}{c} L=-\frac{1}{m}\sum_{i=1}^{m}\log\frac{e^{f_{y1}}}{\sum_{j=1}^{n}e^{f_{j}}}=-\frac{1}{m}\sum_{i=1}^{m}\log\frac{e^{W_{yi}^{T}x_{i}+b_{yi}}}{\sum_{j=1}^{n}e^{W_{yi}^{T}x_{i}+b_{j}}}, \end{array}$$

where *x*_*i*_ represents the *i*^*th*^ HLA distributed simulation evaluation efficacy image feature; *y*_*i*_ represents the real category label corresponding to *i*^*th*^ HLA distributed simulation evaluation efficacy image; *W*_*j*_ represents the category weight; *b*_*j*_ represents the error value of the category; *m* and *n* represent the number of training samples and the number of categories in turn; and *f*_*j*_ represents the inner product relationship *f*_*j*_=*W*_*j*_^*T*^*x*_*i*_+*b*_*j*_ between the category weight *W*_*j*_ and the bias value *b*_*j*_ when the fully connected layer is activated. The HLA distributed simulation evaluation efficacy identification technology should satisfy the following conditions in the HLA distributed simulation evaluation efficacy feature value: the distance between the same HLA distributed simulation evaluation efficacy features needs to be minimized, and the distance between different HLA distributed simulation evaluation efficacy features needs to be maximized.

The corresponding sequential marking algorithm is used to mark the overlapping regions, which cannot ensure the sequential arrangement, so the process of merging the regions will be more complicated. Therefore, this paper proposes a reverse merging algorithm for this limitation. That is, in the process of reverse merging, the processing is performed from the second-to-last overlapping row, and each time a row is processed; the corresponding thread will analyze and correspond to determine whether the merging process is required.

In view of the limitations of existing region-matching algorithms, this paper constructs a parallel region-matching algorithm for HLA distributed simulation, which integrates mobile matching and parallel computing methods and divides the tasks of multiple mobile matching in the simulation into different cores, and the multi-threading methods are used to achieve specific parallelized calculation of region matching, so as to improve the calculation of region-matching ability. Similarly, in each specific thread calculation, the method of using a mobile region to match the region is realized, to reduce the number of redundant calculations and improve the actual efficiency of matching.

**Figure 6:**
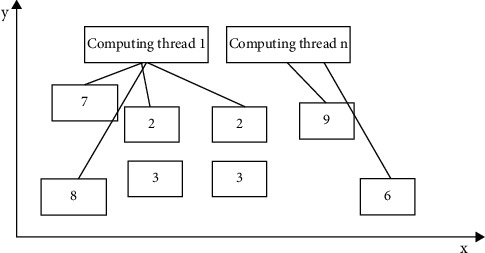
Schematic diagram of the intersecting changes of regional movement.

For the parallel region-matching knowledge recognition algorithm, its specific principles are as follows. First, when a specific single region is effectively moved, the overlap change between other regions and the part of the region is actually related to the movement of the single region. Therefore, when updating a specific region, there is no need to consider the range outside the actual region movement region. The overlap of these regions cannot be directly changed; in the specific simulation process, there is certain irrelevance in regions of the same type. The changes in multiple regions of the same type are overlapped by multiple calculations, as shown in Figure 6.

Figure 6 shows the changes in the overlap of the two-dimensional region during the specific simulation advancement process. When a specific change occurs in the corresponding region, a specific update region can be realized through the boundary of the region, and specific overlapping parallel calculations can be realized from the specific region, and the parallel computing performance can be effectively realized.

When a specific region changes to a certain extent, the relevant core of the matching calculation is caused according to the region, and the position query before and after the movement is realized to ensure the region where the region moves. Here, the dimensional data stored in the region are realized in an orderly manner through a specific index, that is, the data of each dimension in a specific multi-dimensional space are used along with the index for storage. On the one hand, it uses the indexed ordered table to store the specific update region data. On the other hand, it is used to store the range boundary value of various region projections in specific dimensions. Each group is combined through two specific ordered tables, one index table stores the closed point of lower boundary of the range, and the other index table is used to store the opening point of the upper boundary. The definition of the specific data structure of the node is as follows:

  struct Node {
  int id; //range id
  Struct Node ∗ Next; //Node pointer
  }

**Figure 7:**
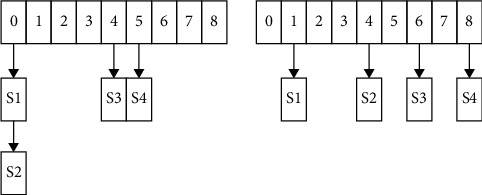
Range representation based on indexed ordered list.

The ordered list of each index implements a specific pointer array index, uses the size of the array to set the dimension, and compares the boundary value with the corresponding index value of the array element, as shown in Figure 7.

It can be seen from Figure 7 that for fixed nodes with no moving range, redundant operation analysis that is not within the operating range can be achieved without effective matching, which effectively saves related computing resources and can achieve accurate analysis and matching in specific ranges, and there is no specific false connection problem.

**Figure 8:**
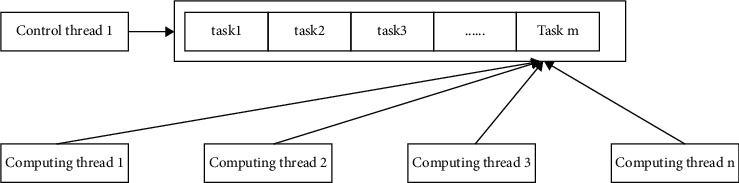
Parallel region-matching method framework.

From the framework of Figure 8, in view of multi-threading, the calculation of overlapping regions is realized, including control threads and multiple computing threads, to complete the calculation task through the task queue. After a specific movement range occurs in the control thread region, it is responsible for the specific task generation node, and it is listed in the specific task queue, and the calculation of the result is completed by the calculation of the control thread. In each simulation advancement process, the first thing that each computing thread needs to process is the update region, until all tasks are processed. The semaphore mechanism is used to synchronize between the threads.

The specific steps of the so-called control thread algorithm are as follows.

For (every simulation advancement process)

1. For each announcement range *a*_*m*_, if *a*_*m*_ moves, insert *a*_*m*_ into the announcement task queue pq.
2. For each order range *b*_*n*_, if *b*_*n*_ moves, insert *b*_*n*_ into the order task queue sq.
3. If pq is not null, for each computing thread 1, release the semaphore m_publish_i, which is used to notify the computing thread to start processing and update the task queue.
4. Wait for the completion semaphore m_publish_finish_i of each computing thread *i* until all update tasks are processed.
5. If sq is not null, for each calculation line *i*, release the semaphore m_subscribe_i, which is used to notify the calculation thread to start processing the order task queue.
6. Wait for the completion semaphore m_subscribe_finish_i of each calculation thread *i* until all the subscription overlap calculations are completed.
7. end for

#### 1.1.3. Simulation Experiment

In order to verify the effectiveness of the parallel region-matching knowledge recognition algorithm, the following simulation experiments are set up in this paper. First, a specific two-dimensional space is generated according to specific user input, and a specific publishing region and ordering region are generated in the two-dimensional space, and corresponding algorithm comparison experiments are designed.

**Figure 9:**
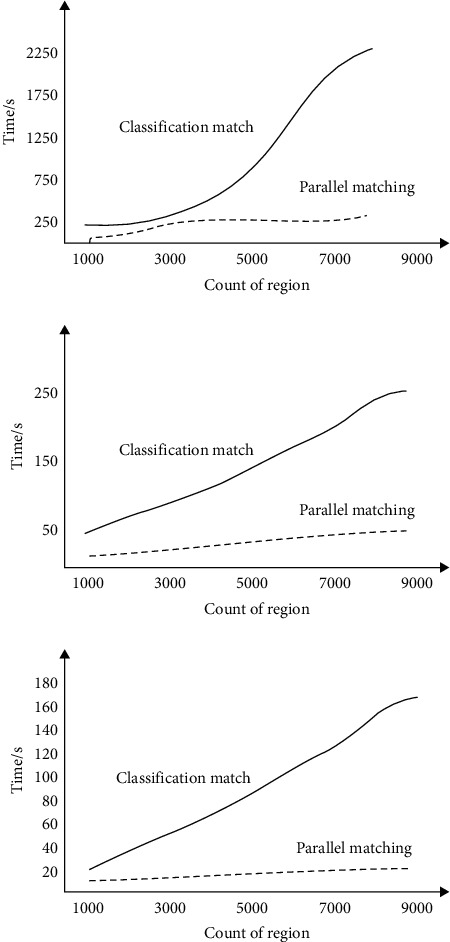
Algorithm performance test results for different numbers of regions (2000, 4000, 8000).

Corresponding tests are carried out for the algorithm performance in different numbers of regions, and the HLA distributed simulation evaluation changes of the matching algorithm in the corresponding cases of 2000, 4000, and 8000 are calculated, respectively, as shown in Figure 9. It can be seen from the results that the parallel region-matching knowledge recognition algorithm is relatively appropriate in terms of time consumption.

**Figure 10:**
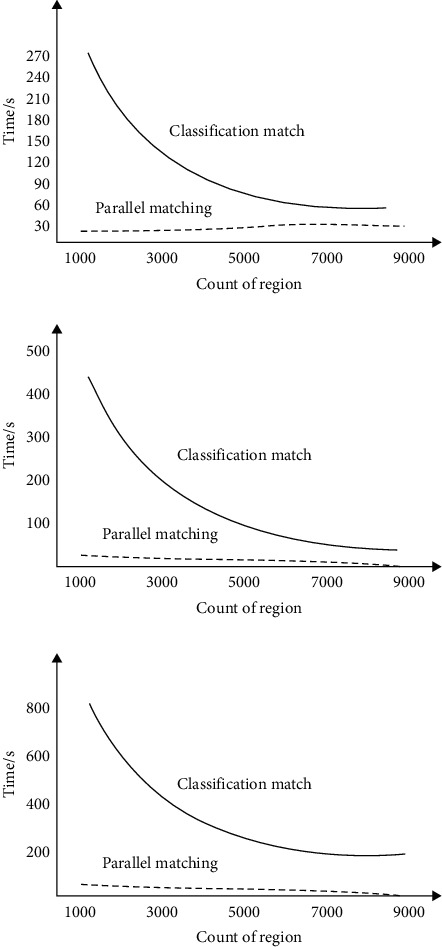
Algorithm performance test results at different dimensional upper limits.

For different upper dimensional limits, as shown in Figure 10, the performance of parallel region-matching knowledge recognition algorithm is obviously better than other algorithms. When the upper limit of dimensionality is greater than 5000, the algorithm performance is the same as the grid matching performance with a grid number of 100, and there is no false connection phenomenon in grid matching.

The parallel region-matching knowledge recognition algorithm is closely related to the number of threads of calculation. From the corresponding simulation experiment results, compared with the traditional method, the parallel region-matching knowledge recognition algorithm does not change the performance with the increase of the specific number of regions, but in terms of specific dimensions, it has a change relationship with the specific upper limit of the dimension. Through multi-core computing power, better acceleration ratio is realized, especially for large-scale regional changes. Therefore, the parallel region-matching knowledge recognition algorithm is more suitable for HLA distributed simulation evaluation.

## 2. Conclusion

The development of industry requires HLA distributed simulation to provide specific support for the long-term, stable, and healthy development of industrial economy. Most of the existing region-matching algorithms need to match all regions, resulting in a waste of computing resources. At the same time, it is difficult to give full play to the parallel computing advantages of multi-core platform mainly based on the idea of serial matching. In view of these needs and shortcomings, based on the parallel region-matching knowledge recognition algorithm, this paper realizes the parallelism of region-matching calculation through multi-core platform. At the same time, for HLA distributed simulation, this paper constructs a simulation system to realize multi-region parallel matching. In the matching calculation, the mobile crossover theory is used to realize the relevant historical information of regional prepositions or backtracking, and it is limited to a fixed threshold range to reduce invalid operations. It has efficient matching and good acceleration performance and can support HLA distributed simulation evaluation. The experimental results show that the algorithm has high matching efficiency, does not cause false connections, and has good acceleration performance. It can give full play to the computing performance of multi-core computing platform and meet the needs of large-scale distributed simulation data distribution and management.

## Data Availability

The data used to support the findings of this study are available from the corresponding author upon request.

## References

[1] Sauter J., Solloch U. V., Giani A. S., Hofmann A J., Schmidt A. H. (2016). Simulation shows that HLA-matched stem cell donors can remain unidentified in donor searches[J]. *Scientific Reports*.

[2] Park Y., Min D. (2015). HLA-DDS API transformation for DDS communication based HLA simulation. *Advanced Science Letters*.

[3] Bouteraa Y., Abdallah I. B., ElMogy A., Ibrahim A., Tariq U., Ahmad T. (2020). A fuzzy logic architecture for rehabilitation robotic systems. *International Journal of Computers, Communications & Control*.

[4] Falcone A., Garro A., Taylor S. ., Anagnostou A.. .. (2017). Experiences in simplifying distributed simulation: the HLA development kit framework. *Journal of Simulation*.

[5] Jung-Yong S., Soumg H. (2016). An implementation of HLA standard for weapon system simulation[J]. *Communications of the Korean Institute of Information Scientists & Engineers*.

[6] Feng X., Gao J. (2019). Gene sequences parallel alignment model based on multiple inputs and outputs. *International Journal of Computers, Communications & Control*.

[7] Wu Y., Gong G. (2015). A real-time scheduling algorithm for HLA-based simulation models[J]. *International Journal of Modeling Simulation and Scientific Computing*.

[8] Gorecki (2018). Integrating HLA-based distributed simulation for management science and BPMN. *IFAC-PapersOnLine*.

[9] Wang Q., Li-Rong A. I, Gong A. Z. (2019). Study on load balancing in hla-based distributed simulation system. *Computer Technology and Development*.

[10] Roth C., Sander O., Kühnle M., Becker J. (2013). Hla-based simulation environment for distributed systemc simulation. *Ai Trade News*.

[11] Guo D., Zhang J., Liang Q. (2019). Design of distributed training simulation system based on hla. *Radio Engineering*.

[12] Sato C., Mio K., Ogura T. (2017). 3P-031 molecular dynamics simulation studies on the differences in the binding mechanism of LILRB1/HLA-G and LILRB2/HLA-G(Protein:Structure & Function,The 47th annual meeting of the biophysical society of Japan)[J]. *Seibutsu Butsuri*.

[13] Nowroozi A., Shahlaei M. (2016). A coupling of homology modeling with multiple molecular dynamics simulation for identifying representative conformation of GPCR structures: a case study on human bombesin receptor subtype-3. *Journal of Biomolecular Structure and Dynamics*.

[14] Kwok J., Guo M., Yang W. (2018). Simulation of non-inherited maternal antigens acceptable HLA mismatches to increase the chance of matched cord blood units: Hong Kong’s experience. *Human Immunology*.

[15] You Y., Tan L., Lee T., Kim W., Yoon S. (2016). Development of an OMT table viewer/editor using the ms-based distributed simulation. *International Journal of Information and Electronics Engineering*.

[16] Albagli A. ., Falcão D. ., de Rezende J. . (2016). Smart grid framework co-simulation using HLA architecture. *Electric Power Systems Research*.

[17] Tafulo S, Malheiro J, Dias L (2020). Improving HLA matching in living donor kidney transplantation using kidney paired exchange program. *Transplant Immunology*.

[18] Lee S., Lee S., Hwang K., Kim S. (2015). Design and implementation of the multi-resolution interoperation simulation using HLA/RTI. *Journal of the Korea Society for Simulation*.

[19] Ozbek P. (2016). Dynamic characterization of HLA-B∗44 Alleles: a comparative molecular dynamics simulation study. *Computational Biology and Chemistry*.

[20] Kwok J., Tang W. H., Chu W. K. (2020). High resolution allele genotyping and haplotype frequencies for NGS based HLA 11 loci of 5266 Hong Kong Chinese bone marrow donors[J]. *Human Immunology*.

[21] Rad F. R., Akbari M. G., Zamani M., Bayat S., Zamani M. (2021). Pharmacogenetic and association studies on the influence of HLA alleles and rivastigmine on the Iranian patients with late-onset alzheimer’s disease. *Molecular Neurobiology*.

[22] Shin B. H., Everly M. J., Zhang H. (2020). Impact of tocilizumab (Anti–IL-6R) treatment on immunoglobulins and anti-HLA antibodies in kidney transplant patients with chronic antibody-mediated rejection[J]. *Transplantation*.

[23] Chatterjee D., Priyadarshini P., Das D. ., Mushtaq K.., Agrewala J. . (2020). Deciphering the structural enigma of HLA class-II binding peptides for enhanced ipve. *Journal of Proteome Research*.

[24] Arévalo M., López-Medina C., Martinez-Losa M. M. (2020). Role of HLA-B27 in the comorbidities observed in Axial Spondyloarthritis: data from COMOSPA. *Joint Bone Spine*.

[25] Hernández-Hernández A., Hernández-Zaragoza B., Barquera R. (2020). Genetic diversity of HLA system in two populations from Oaxaca, Mexico: oaxaca city and rural Oaxaca - ScienceDirect. *Human Immunology*.

[26] Liu Y., Sun H., Fan W., Xiao T. (2015). A parallel matching algorithm based on order relation for HLA data distribution management. *International Journal of Modeling Simulation and Scientific Computing*.

[27] Abed A., Calapre L., Lo J. (2020). 301MO Genomic HLA as a predictive biomarker for survival among non-small cell lung cancer patient treated with single agent immunotherapy. *Annals of Oncology*.

[28] Fainardi E., Bortolotti D., Castellazzi M., Casetta I., Bellini T., Rizzo R. (2020). Detection of serum soluble HLA-G levels in patients with acute ischemic stroke: a pilot study - ScienceDirect. *Human Immunology*.

